# Adalimumab Increases Follicle Reserve and Follicle Development in Rat Ovary: The Effect of Adalimumab on Ovarian Reserve

**DOI:** 10.7759/cureus.11230

**Published:** 2020-10-29

**Authors:** Selçuk Kaplan, Ahmet Türk

**Affiliations:** 1 Obstetrics and Gynaecology, Adıyaman University Faculty of Medicine, Adıyaman, TUR; 2 Histology and Embryology, Adıyaman University Faculty of Medicine, Adıyaman, TUR

**Keywords:** adalimumab, ovarian follicle reserve, ovarian toxicity, rat, rheumatoid arthritis

## Abstract

Introduction

Adalimumab is used in the treatment of many chronic inflammatory diseases, especially rheumatoid arthritis. The aim of this experimental study is to determine the histological and biochemical effects of adalimumab on rat ovary.

Methods

Wistar albino female rats were randomly divided into three groups prior to the experiment: a healthy control group, a 2 mg/kg adalimumab group, and a 5 mg/kg adalimumab group. Then, histopathological findings and biochemical examinations were made in the ovaries of the rats. Hematoxylin-eosin staining, morphometric examination, and Masson trichrome staining were performed. Antimullerian hormone (AMH) levels were measured in the biochemical examination.

Results

Ovarian follicle count and AMH level were significantly higher in the groups given low-dose adalimumab and high-dose adalimumab (p <0.001). In addition, fibrosis decreased in proportion to the dose of adalimumab (p <0.001).

Conclusion

Adalimumab is an important biological agent that contributes to the preservation of ovarian function by increasing ovarian follicle reserve and has shown that it can help preserve ovarian reserve in women of reproductive age suffering from chronic inflammatory diseases.

## Introduction

Rheumatoid arthritis (RA) is a chronic inflammatory disease that is common in women of reproductive age. Subfertility (pregnancy duration longer than 12 months despite regular unprotected intercourse) is a common problem in women with RA; approximately 40% of women with RA suffer from subfertility [1-3]. Women with RA are more often nulliparous than healthy women [4]. Among patients with RA, subfertility has been associated with the use of non-steroid anti-inflammatory drugs (NSAIDs) and prednisone [2]. However, little is known about how biological agents, which have been frequently used in patients with RA in recent years, affect ovarian function and fertility. In addition, the low age of menopause in women with RA has been evaluated in relation to premature ovarian failure [5-6]. In a recent study, it was shown that ovarian reserve decreases in women with RA [7].

Many important markers, such as menstrual cycle pattern, primordial ovarian count, hormonal value (follicle-stimulating hormone (FSH), luteinizing hormone (LH), and estradiol), and anti-mullerian hormone (AMH) are used to determine ovarian reserve [8]. In histological examinations, the number of follicles with hematoxylin-eosin staining and the primordial follicle reserve are important parameters for determining ovarian reserve [9]. AMH is produced by the granulosa cells of preantral and antral follicles and is one of the best ovarian reserve markers independent of the menstrual cycle [10]. Moreover, AMH can be used to indicate ovarian reserve after gonadotoxic therapy such as chemotherapy, radiotherapy, or surgery.

Adalimumab, a fully human immunoglobulin (Ig) G1 neutralizing monoclonal antibody specific to tumor necrosis factor-α (TNF-α) is used in the treatment of many chronic inflammatory diseases, especially RA [11-12]. A recent study evaluating ovarian reserve in patients with juvenile RA using ultrasonographic examination and AMH, LH, and FSH levels showed that ovarian functions were not affected in patients using biological agents such as adalimumab and etanercept [13]. However, there are very few studies on this subject. As such, the aim of this experimental study was to determine the histological and biochemical effects of adalimumab on rat ovaries.

## Materials and methods

The experiments in this study were approved by our local animal ethics committee and carried out in accordance with the National Institutes of Health animal research guidelines (Date: 04.06.2020, Ethics committee number: 2020/022).

Animals and experimental protocol

In the present study, 21 10- to 12-week-old Wistar albino female rats, weighing 250-280 g, were divided into three groups of seven animals each. To enable adaptation, the animals were placed in the cage for seven days without treatment at room temperature 22 ± 20°C, with 12 hours of dark and 12 hours of light, and water and feed. Similar conditions were maintained throughout the experiment.

Sixty mg/kg ketamine hydrochloric acid (Ketalar Eczacibasi Warner-Lambert Pharmaceutical, Istanbul, Turkey) and 7 mg/kg xylazine hydrochloric acid (Rompun, Bayer, Istanbul, Turkey) were administered intramuscularly to all rats as anesthetics. For Group 1 (control group) (n = 7), after the adnexa was localized with the right and left uterine horns, the abdominal wall was closed with 4-0 nylon sutures. No other actions were applied during the experiment. For Group 2 (low-dose adalimumab group) (n = 7), after the adnexa was localized with the right and left uterine horns, the abdominal wall was closed with 4-0 nylon sutures, and 2 mg/kg adalimumab intraperitoneal was given every day for four weeks. For Group 3 (high-dose adalimumab group) (n = 7), after the adnexa was localized with the right and left uterine horns, the abdominal wall was closed with 4-0 nylon sutures, and 5 mg/kg adalimumab intraperitoneal was given every day for four weeks.

Histopathological evaluation

Hematoxylin and Eosin Staining and Follicular Classification

Ovarian tissues were fixed with 10% formaldehyde and embedded in paraffin blocks after a routine tissue placement procedure. Then, 4-6 μm sections were obtained from the paraffin blocks and stained with hematoxylin and eosin (H&E), and the prepared samples were photographed by counting the follicles using a Leica DM500 microscope (Leica Microsystems, Wetzlar, Germany) [14]. In accordance with a previous study [15], the follicles were classified as follows: primordial follicle (an oocyte surrounded by a flattened single layer of granulosa cell), a primary follicle (an oocyte surrounded by a cuboidal single layer of granulosa cell), a secondary follicle (multiple layers of cuboidal granulosa cells and an oocyte without antral space), and an antral follicle (oocyte with antral space or multiple (four) layers of granulosa cells). Oocytes with a fragmented nucleus, degenerated oocytes, or antral follicles with irregular granulosa cells were considered atretic [16]. Each follicle with the above-mentioned characteristics was counted once, and groups were formed according to the number of follicles.

Masson’s Trichrome Staining

Masson’s trichrome dye was used for fibrosis grading and evaluated semi-quantitatively. First, the 4 µm deparaffinized sections were stained with an acid dye (acid fuchsin). Then, they were treated with phosphomolybdic acid, and, finally, they were stained with methyl blue. Old collagen fibers were dyed red, and the newly formed collagen fibers were blue. Accordingly, the following scale was used: 0 = no fibrosis, +1 = low fibrosis, +2 = moderate fibrosis, and +3 = severe fibrosis [17].

Biochemical examination

Assay of Plasma AMH Concentrations

The AMH levels (ng/mL) of the serum samples were determined by the Enzyme-Linked ImmunoSorbent Assay (ELISA) method (Thermo Scientific Multiskan GO, Finland) using commercially available rat ELISA kits. All samples were tested using the same assay. The sensitivity of the assay was 0.10 ng/mL, and the assay ranges were 0.3-40 ng/mL.

Statistical analysis

Statistical analysis was performed using SPSS® Statistics 22 for Windows software (IBM Corp., Armonk, NY). The Shapiro-Wilk test was used as a normal distribution test. The one-way analysis of variance (ANOVA) test (the post-hoc test Bonferroni test) was used to analyze data that conformed to the normal distribution. The chi-square test was used to analyze the qualitative data, and p < 0.05 was considered significant.

## Results

Histopathological results

Hematoxylin-Eosin Staining and Morphometric Examination Results

**Figure 1:**
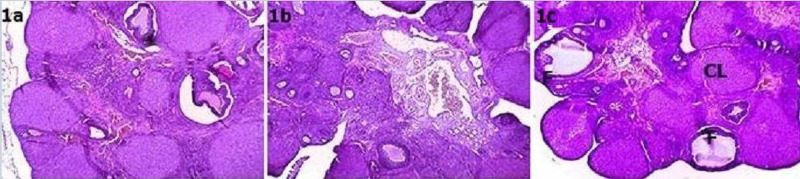
1a-1c: Hematoxylene-eosin over tissues 4X10 1a: Control group; 1b: Low-dose adalimumab group; 1c: High-dose adalimumab group

Based on an evaluation of the H&E staining, the total number of follicles was increased in the low- and high-dose adalimumab groups than in the control group. While there were statistically significant differences between the numbers of primordial, primary, secondary, and antral follicles in the groups, there were no significant differences between the corpus luteum numbers (Table 1, Figure 1).

**Table 1:** Number of follicles by groups SD: Standard deviation; LD ADA: Low-dose adalimumab group; HD ADA: High-dose adalimumab group

Group		Primordial Follicle	Primary Follicle	Secondary Follicle	Antral Follicle	Corpus Luteum
Control Group	Mean±SD	5,96±0,23	3,12±0,13	6,99±0,66	1,20±0,037	19,88±0,43
	Median	6,01	3,14	7,03	1,20	20,02
LD ADA	Mean±SD	6,90±0,41	4,05±0,14	8,88±0,23	1,28±0,042	15,69±0,96
	Median	7,09	4,10	9,02	1,28	18,01
HD ADA	Mean±SD	10,91±1,06	4,92±0,20	14,28±1,32	2,10±0,30	13,56±0,89
	Median	11,10	4,90	14,90	2,21	15,60
	p	<0.001	<0.001	<0.001	<0.001	0.063

Masson Trichrome Staining Results

**Figure 2:**
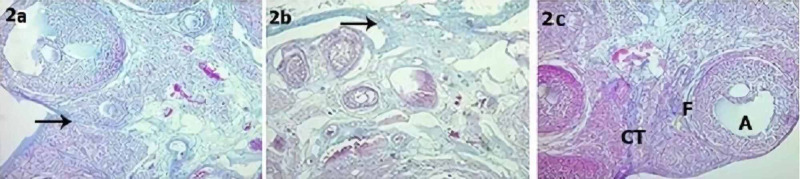
2a: Control group; 2b: Low-dose adalimumab group; 2c: High-dose adalimumab group CL: Corpus luteum, F: Follicle, CT: Connective tissue, A: Antrum, Black arrow: Connective tissue increase

There was a significant difference in fibrosis severity between the groups (Table 2, Figure 2). While 14.3% of the high-dose adalimumab group had mild fibrosis, 28.6 % of the control group had mild fibrosis, the low-dose adalimumab group 57.1% had moderate fibrosis, and 42.9% had severe fibrosis (Table 2). Fibrosis severity was lower in the low- and high-dose adalimumab groups than in the control group and lower in the high-dose adalimumab group than in the low-dose adalimumab group (p < 0.001).

**Table 2:** Comparison of fibrosis severity according to experimental groups ADA: Adalimumab

			Fibrosis				p
			None	Low	Mild	Severe	
Group	Control Group	n	0	1	3	3	
		%	0,0	14,3	42,9	42,9	
	Low-Dose ADA	n	0	4	3	0	
		%	0,0	57,1	42,9	0,0	
	High-Dose ADA	n	6	1	0	0	
		%	85,7	14,3	0,0	0,0	<0.001

**Figure 3:**
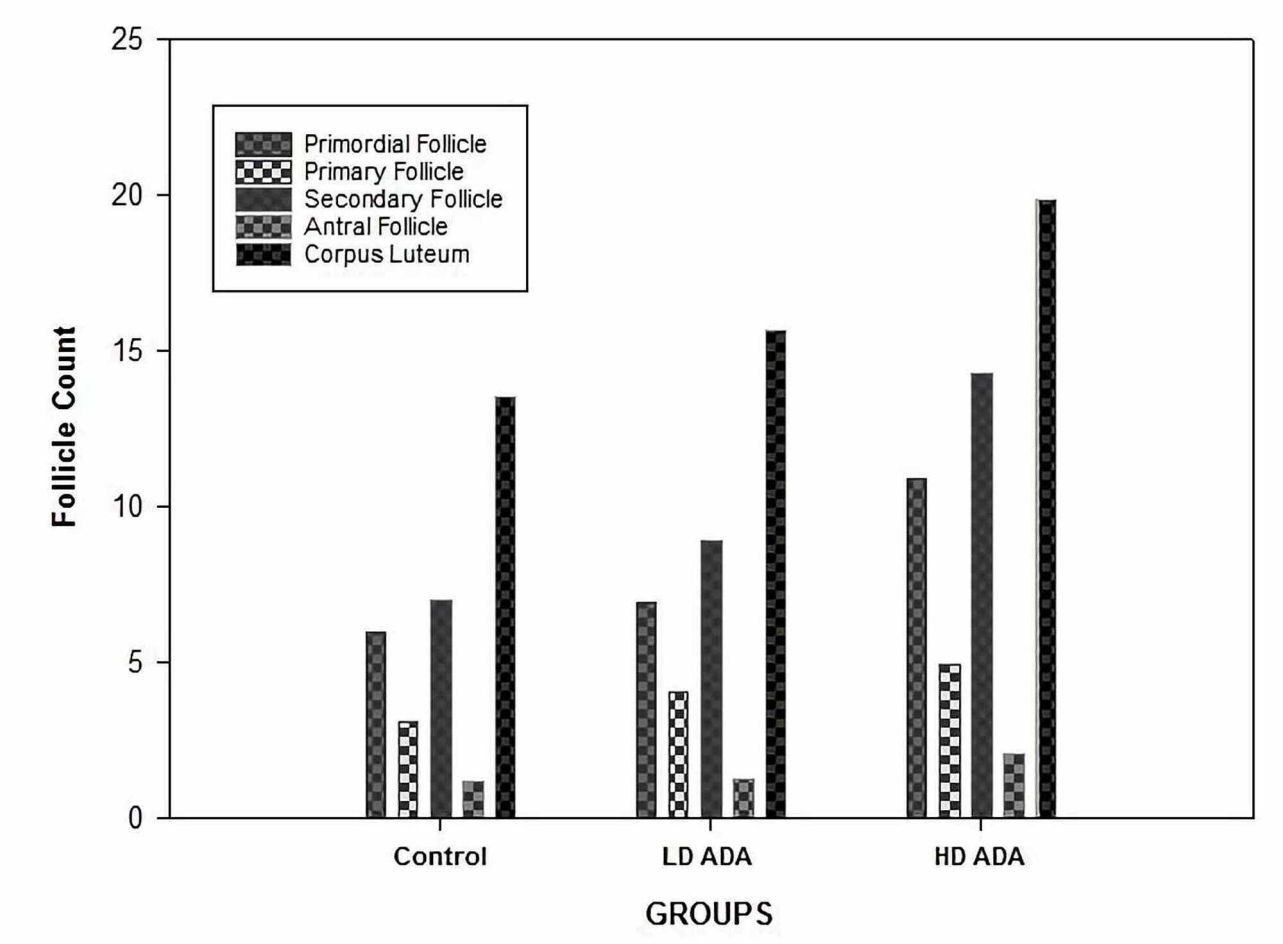
Graphic showing the number of follicles according to the experimental groups

The post-hoc analysis of the differences in follicle numbers between the groups is shown in Table 3. The number of primordial follicles was larger in the low- and high-dose adalimumab groups as compared to the control group (p < 0.001). In addition, the number of primordial follicles was larger in the high-dose adalimumab group than in the low-dose adalimumab group (p = 0.043). In addition, primary, secondary, and antral follicle numbers were higher in the low- and high-dose adalimumab groups than in the control group (p < 0.001). Moreover, the primary follicle number (p < 0.001) and the secondary follicle number (p = 0.002) were larger in the high-dose adalimumab group than those in the low-dose adalimumab group (Table 3, Figure 3).

**Table 3:** Post-hoc analysis of experimental groups ADA: Adalimumab, LD: Low dose, HD: High dose

Dependent Variable	Group	Group	Mean Difference	p
Primary Follicle	High-Dose ADA	Control	1,79^*^	<0,001
		LD ADA	0,87^*^	<0,001
	Control	HD ADA	-1,79^*^	<0,001
		LD ADA	-0,92^*^	<0,001
	Low-Dose ADA	HD ADA	-0,87^*^	<0,001
		Control	0,92^*^	<0,001
Secondary Follicle	High-Dose ADA	Control	7,29^*^	<0,001
		LD ADA	5,39^*^	<0,001
	Control	HD ADA	-7,29^*^	<0,001
		LD ADA	-1,89^*^	0,002
	Low-Dose ADA	HD ADA	-5,39^*^	<0,001
		Control	1,89^*^	0,002
Primordial Follicle	High-Dose ADA	Control	4,95^*^	<0,001
		LD ADA	4,00^*^	<0,001
	Control	HD ADA	-4,95^*^	<0,001
		LD ADA	-,94^*^	0,043
	Low-Dose ADA	HD ADA	-4,00^*^	<0,001
		Control	,94^*^	0,043
Antral Follicle	High-Dose ADA	Control	0,90^*^	<0,001
		LD ADA	0,81^*^	<0,001
	Control	HD ADA	-0,90^*^	<0,001
		LD ADA	-0,08	1,000
	Low-Dose ADA	HD ADA	-0,81^*^	<0,001
		Control	0,08	1,000
* groups with differences				

Biochemical evaluation

AMH Level

**Figure 4:**
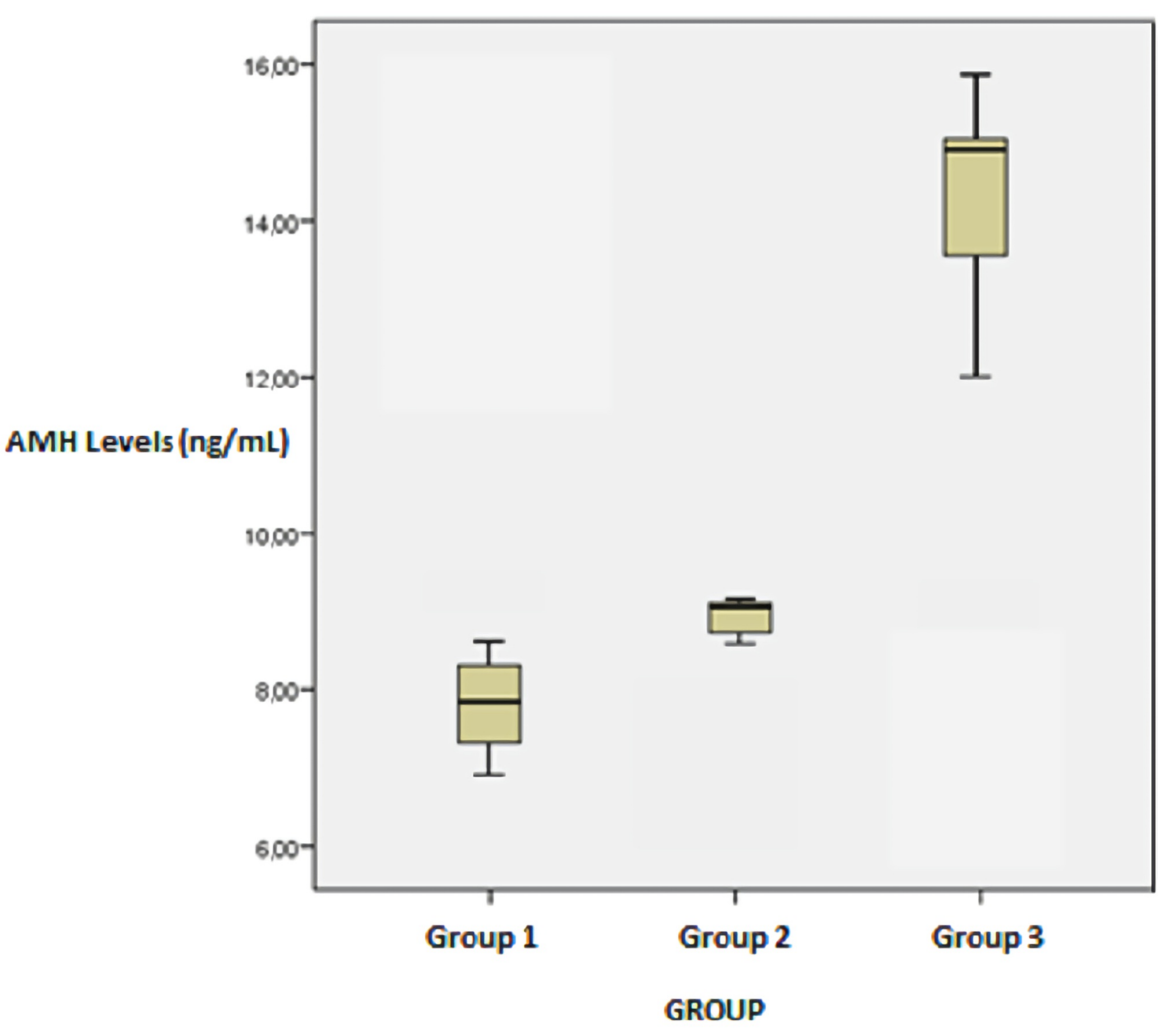
Box-plot graph showing AMH levels according to experimental groups AMH: Antimullerian hormone

After adalimumab administration, the mean AMH level increased in both groups as compared to the control group (Table 4). The AMH level increased in the high-dose adalimumab group as compared to the low-dose adalimumab group (p = 0.007). Total follicle number and AMH values were correlated in the high-dose adalimumab group (p = 0.041). However, there was no statistically significant difference in other groups (p > 0.05) (Figure 4).

**Table 4:** Comparison of AMH values according to experimental groups ADA: Adalimumab; AMH: Antimullerian hormone; * p=0.007, ** p=0.041

	Control Group	Low-Dose ADA	High-Dose ADA
AMH (ng/mL)	6.14±0.39	9.83±0.51	13.71±0.62*
Total Follicle	17.27±0.74	21.11±1.13	32.21±2.72**

## Discussion

The biochemical and histopathological findings in our study showed that adalimumab increased the number of follicles in the ovary and decreased fibrosis. Meanwhile, the AMH level, which is a valuable marker showing follicle reserve, increased in rats given a high dose of adalimumab.

The inflammation process is related to the production of cytokines and chemokines and the associated damage caused by leukocytes. TNF-α is an important cytokine that plays a role in ovarian ischemia-reperfusion injury. An increase in TNF-α levels in the ovaries causes leukocyte infiltration and leads to ovarian tissue damage and a decrease in ovarian reserve [18]. Another study investigating the effects of the biological agent etanercept on an ovarian ischemia-reperfusion injury showed that etanercept reduced injury effect on ovarian damage [19]. Many studies have shown that TNF-α inhibitors improve infertility treatment outcomes [20-21]. In our study, the increase in ovarian reserve in rats given adalimumab can be explained in light of this information. AMH is a marker used for preantral follicle reserve in the ovarian pool [22] and is secreted from granulosa cells of antral follicles [10]. Therefore, it is not coincidental that the increase in AMH, another marker showing an increase in ovarian reserve, was higher in the high-dose adalimumab group. In women with recurrent pregnancy loss, an increase in the T helper type 1 (Th1) cell count in peripheral blood lymphocytes has been found [23]. Furthermore, an increase in the Th1/Th2 ratio may cause implantation failure and spontaneous abortion [24]. TNF-α inhibitors increase the success of in vitro fertilization by changing the Th1/Th2 balance [21]. In a study of 25 RA patients who received anti-TNF-α inhibitor therapy, the spontaneous pregnancy duration was shortened in women treated with biological agents [25]. The increase in ovarian follicle reserve in rats given adalimumab in our study supports these publications.

Ovarian fibrosis plays an important role in the physiological and pathological processes of the ovaries. A fibrotic focus in the ovary seriously affects reproductive health and quality of life in women. Ovarian fibrosis may cause premature ovarian failure by reducing ovarian functions [26]. It has been reported that follicular atresia, which causes ovarian dysfunction, may be associated with increased TNF-α and other proinflammatory cytokines in rats with experimentally developed polycystic ovary [27-29]. In this study, we performed laparotomy for ovarian fixation in all groups. While fibrosis developed secondary to this operation in the control group, fibrosis decreased in proportion to the dose of adalimumab. This effect of adalimumab may be related to the inhibition of TNF-α by adalimumab. This activity may suggest that it may be a treatment option for early recovery after pelvic surgeries and for women suffering from premature ovarian insufficiency.

## Conclusions

Adalimumab is an important biological agent that contributes to the preservation of ovarian function by increasing ovarian follicle reserve. This study demonstrated that it may help preserve ovarian reserve in women of reproductive age suffering from chronic inflammatory diseases. As a result, the findings of our study should be supported by a larger sample of cases and human studies.
